# Comment on “Intramolecular Proton-Coupled Hydride Transfers with Relatively Low Activation Barriers”

**DOI:** 10.1021/acs.jpca.5c05958

**Published:** 2025-12-12

**Authors:** Vinicius Martinelli, Wagner Eduardo Richter

**Affiliations:** Department of Chemistry, 42487 State University of Maringá, Maringá, 87020-900 PR, Brazil

Karton and co-workers have recently reported on the mechanism for the intramolecular proton-coupled hydride transfer (PCHT) in bifunctional molecules containing carbonyl and hydroxyl groups.^1^ The authors argued that two oppositely charged hydrogen atoms (H^δ+^ and H^δ−^) are transferred simultaneously. This assignment was primarily supported by “APT charges” derived from theoretical calculations. In this comment, we aim to explore the H^δ+^ and H^δ−^ assignment (also called H_p_ and H_h_, for *proton* and *hydride*, respectively) using a broader set of charge models, as well as to address a specific feature from the “APT charges” (here called GAPT, see below) that makes them unsuitable for such application.

Let us first explore the H_p_ and H_h_ assignments for the hydrogens being transferred within the reaction. Karton et al.^1^ have tested 36 different transition state structures and used only GAPT (called by Karton et al. “APT charge”) as the charge model, while works from Cho et al.^2^ and Manz^3^ report more than 20 different charge models available in the literature. Aiming at the simplest and more concise discussion possible, and with as few calculations as necessary, we selected eight charge models to be used on Karton’s systems with *n* = 1, 2 and two substituent configurations, R_1_ = R_2_ = H and R_1_ = H; R_2_ = Me. Even though we use here a smaller test set, the conclusions taken from it can be readily extended to any other molecule, if desired.

Starting with the transition state structures (TSSs) for R_1_ = H; R_2_ = Me; *n* = 1, 2 available at their Supporting Information,^1^ we built the respective R_1_ = R_2_ = H structures (*n* = 1, 2) and optimized all of them at the same level of theory (B3LYP/6-31G­(2df,p)) reported therein, followed by a vibrational analysis at the same level as well. This confirmed that all the structures were true stationary points in the potential energy surface (*xyz* coordinates and imaginary frequencies available at the Supporting Information). On these reoptimized structures, we calculated atomic charges using NPA,^4^ CHELPG,^5^ CM5,^6^ Hirshfeld,^7,8^ QTAIM,^9–11^ ADCH,^12^ VDD (charges from Voronoi Deformation Densities)^13^ and Becke partition schemes. Table 1 shows the atomic charges calculated for the atoms involved in the double hydrogen transfer in these TSSs (following the same labeling as given by Karton et al., see Figure 1). The GAPT parameter is also shown, but we recall it does not come from a standard population analysis, but rather from vibrational analysis.^14^

**Figure 1:**
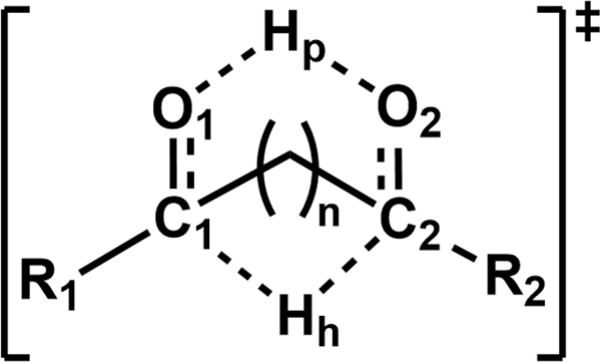
Simplified scheme for proton-coupled hydride transfer, featuring the atom labeling used in the following tables.

**Table 1:** Atomic Charges for the Transition State Structures of **n1**_**HH**, **n2**_**HH**, **n1**_**HMe**, and **n2**_**HMe**, Using NPA, ChelpG, CM5, ADCH, Voronoi, Becke, Hirshfeld and QTAIM Partition Schemes^a^

**n1_HH**	GAPT	NPA	ChelpG	CM5	ADCH	VDD	Becke	Hirshf	AIM
H_h_	–0.295	0.155	–0.116	0.069	0.005	0.030	0.034	0.009	–0.084
H_p_	0.405	0.512	0.403	0.305	0.235	0.195	0.446	0.114	0.586
C_1_	0.668	0.149	0.430	0.050	0.014	0.087	0.183	0.099	0.819
O_1_	–0.680	–0.700	–0.536	–0.375	–0.307	–0.274	–0.508	–0.232	–1.156
C_2_	0.668	0.150	0.433	0.050	0.014	0.089	0.183	0.099	0.818
O_2_	–0.680	–0.700	–0.537	–0.375	–0.307	–0.276	–0.508	–0.232	–1.156

**n2_HH**	GAPT	NPA	ChelpG	CM5	ADCH	Voronoi	Becke	Hirshf	AIM
H_h_	–0.293	0.176	–0.092	0.091	0.041	0.073	0.078	0.018	–0.040
H_p_	0.366	0.514	0.376	0.305	0.264	0.193	0.444	0.108	0.592
C_1_	0.715	0.137	0.353	0.040	0.021	0.073	0.171	0.092	0.799
O_1_	–0.649	–0.688	–0.507	–0.373	–0.323	–0.269	–0.505	–0.223	–1.143
C_2_	0.715	0.137	0.361	0.040	0.021	0.072	0.172	0.092	0.800
O_2_	–0.649	–0.688	–0.508	–0.373	–0.324	–0.269	–0.505	–0.223	–1.143

**n1_HMe**	GAPT	NPA	ChelpG	CM5	ADCH	Voronoi	Becke	Hirshf	AIM
H_h_	–0.340	0.145	–0.142	0.062	0.003	0.026	0.052	0.001	–0.095
H_p_	0.381	0.519	0.395	0.310	0.247	0.197	0.435	0.119	0.614
C_1_	0.728	0.107	0.460	0.024	0.008	0.070	0.164	0.074	0.857
O_1_	–0.742	–0.709	–0.581	–0.394	–0.363	–0.333	–0.551	–0.284	–1.168
C_2_	0.770	0.396	0.612	0.138	0.123	0.120	0.328	0.148	0.776
O_2_	–0.661	–0.697	–0.536	–0.364	–0.275	–0.226	–0.505	–0.192	–1.178

**n2_HMe**	GAPT	NPA	ChelpG	CM5	ADCH	Voronoi	Becke	Hirshf	AIM
H_h_	–0.343	0.179	–0.107	0.091	0.042	0.080	0.096	0.015	–0.044
H_p_	0.366	0.516	0.369	0.305	0.258	0.189	0.441	0.107	0.597
C_1_	0.784	0.127	0.345	0.030	0.016	0.068	0.163	0.083	0.807
O_1_	–0.693	–0.696	–0.520	–0.380	–0.336	–0.288	–0.520	–0.241	–1.146
C_2_	0.808	0.340	0.564	0.113	0.120	0.086	0.316	0.128	0.780
O_2_	–0.674	–0.697	–0.542	–0.372	–0.319	–0.260	–0.544	–0.216	–1.158

a All values are in units of electrons (e).

First of all, it is important to notice that the GAPT values at Table 1 for **n1**_**HMe** and **n2**_**HMe** are in perfect agreement with the values reported by Karton et al. (first two entries in Table 2 of their paper^1^), as expected. From Table 1, it is easy to see that although H_p_ is indeed positively charged according to all the population analysis we have tested, there is no agreement regarding the charge for H_h_. Actually, there are more models predicting it to be positively charged than models predicting it to be negatively charged, even though the positive values in the vast majority of cases are smaller than 0.01*e*. On the other hand, we also see that the model that predicts the most negative charges for H_h_ is indeed GAPT, followed by ChelpG and QTAIM, but the two latter suggest values that are much less negative than GAPT, approaching more neutral charges instead. Therefore, labeling “H^δ−^” the hydrogen atom being transferred along with H_p_ seems to be not an accurate interpretation of the electronic population on these atoms.

Once we know that GAPT does not provide an accurate description of the atomic charges in molecules, we recall why. The literature normalized naming it “APT charge”, but the original proponent suggested the name GAPT (for *Generalized Atomic Polar Tensor*).^15^ This parameter is obtained from the trace of the Atomic Polar Tensor, APT,^16,17^ which is the squared array of all the possible derivatives of the molecular dipole moment with respect to displacement of a given atom:

1 $$P_{A}=\left(\begin{array}{ccc} \frac{\partial p_{x}}{\partial x_{A}} & \frac{\partial p_{x}}{\partial y_{A}} & \frac{\partial p_{x}}{\partial z_{A}}\\ \frac{\partial p_{y}}{\partial x_{A}} & \frac{\partial p_{y}}{\partial y_{A}} & \frac{\partial p_{y}}{\partial z_{A}}\\ \frac{\partial p_{z}}{\partial x_{A}} & \frac{\partial p_{z}}{\partial y_{A}} & \frac{\partial p_{z}}{\partial z_{A}} \end{array}\right)\Rightarrow q_{A}^{GAPT}=\frac{1}{3}\left(\frac{\partial p_{x}}{\partial x_{A}}+\frac{\partial p_{y}}{\partial y_{A}}+\frac{\partial p_{z}}{\partial z_{A}}\right)$$

The molecular dipole moment has dimensions of charge· distance (typically *C*·*m*) whereas the atomic displacements have dimensions of distance (*m*), thus making all the derivatives within the APT have dimensions of *C*·*m*/*m* = *C*, which, in atomic units, are expressed as multiples of the elementary charge, *e*. Note that these derivatives are dynamic (rather than static) parameters as they express rates of change of the dipole when a given atom is displaced from its equilibrium position. Being dynamic rather than static, they should not be used as parameters for static (typically, but not restricted to) equilibrium geometries; unfortunately, this is not the case, and most of its citations use GAPT as if it was a true charge model, and even compare GAPT with other charge models available in the literature.^18–28^

Such misunderstanding regarding GAPT (which we deliberately avoid calling “*APT charge*”) motivated many papers from our group along the past decade.^14,29–31^ Making a long story short, we have demonstrated (with both equations and numerical examples) that GAPT can be divided into smaller terms;^14^ if we model the molecular dipole moment as being dependent solely on the atomic charges (eq 2), one of these terms will be the charge itself (*C*) calculated using one among various partition schemes available in the literature, and the other being related to the changes in these charges that must occur when the atom is displaced (charge transfer, CT). In case we use a partition scheme that also features atomic dipoles (eq 3), changes in the atomic dipoles (dipolar polarization, DP) also need to be considered.^32,33^ Therefore, these changes will also appear in GAPT:^14,30,34^

2 $$\mathrm{p⃗}=\overset{N}{\underset{i=1}{\sum }}\left(q_{i}\cdot {\mathrm{r⃗}}_{i}\right)\Rightarrow q_{A}^{GAPT}=q_{A}^{C}+q_{A}^{CT}$$

3 $$\mathrm{p⃗}=\overset{N}{\underset{i=1}{\sum }}\left(q_{i}\cdot {\mathrm{r⃗}}_{i}+{\mathrm{m⃗}}_{i}\right)\Rightarrow q_{A}^{GAPT}=q_{A}^{C}+q_{A}^{CT}+q_{A}^{DP}$$

As long as a given population analysis successfully reproduces the molecular dipole moment from the wave function, it will also reproduce GAPT. For example, ChelpG^5^ and ADCH^12^ charges can be used to model GAPT (as a C + CT sum), as well as atomic charges and atomic dipoles from QTAIM,^9,10^ Hirshfeld^7,8^ and DDEC6 ^35^ partition schemes (C + CT + DP). On the other hand, Mulliken,^36^ NPA,^4^ CM5 ^6^ and Voronoi^13^ cannot because they do not reproduce the molecular property for an arbitrary geometry (in simple mathematical terms, for Mulliken, NPA, CM5, and Voronoi, *q*_A_^GAPT^ ≠ *q*_A_^C^ + *q*_A_^CT^).^30^ It is worth mentioning that out–of–plane vibrations in planar molecules can only be correctly modeled by CCTDP models, thus showing that including atomic polarizations into these models is not a matter of choice but a necessary feature for correctly studying electronic density distributions subjected to vibrations.^14,30,31,34,37^

We computed these CCT/CCTDP partitions of GAPT for the aforementioned molecules using ChelpG (just atomic charges, no atomic dipoles, leading to a CCT partition) and Hirshfeld and QTAIM atomic charges and atomic dipoles (thus making a CCTDP partition). The partitions for **n1**_**HH** are presented in Table 2, whereas the complete set (for **n2**_**HH**, **n1**_**HMe** and **n2**_**HMe** is presented in Supporting Information. Details on how to perform this analysis can be found elsewhere.^14,29,32^

**Table 2:** ChelpG/CCT, Hirshfeld/CCTDP and QTAIM/CCTDP Partitions for GAPT in **n1**_**HH** ^a^

CHELPG	C	CT	DP	Sum	GAPT	Diff
H_h_	–0.116	–0.180		–0.295	–0.295	0.000
H_p_	0.403	0.002		0.405	0.405	0.000
C_1_	0.430	0.239		0.669	0.668	0.000
O_1_	–0.536	–0.145		–0.680	–0.680	0.000
C_2_	0.433	0.235		0.668	0.668	0.000
O_2_	–0.537	–0.143		–0.680	–0.680	0.000

Hirshfeld	C	CT	DP	Sum	GAPT	Diff
H_h_	0.009	–0.268	–0.036	–0.295	–0.295	0.000
H_p_	0.114	0.242	0.049	0.405	0.405	0.000
C_1_	0.099	0.426	0.144	0.668	0.668	0.000
O_1_	–0.232	–0.338	–0.110	–0.680	–0.680	0.000
C_2_	0.099	0.426	0.143	0.668	0.668	0.000
O_2_	–0.232	–0.338	–0.110	–0.680	–0.680	0.000

QTAIM	C	CT	DP	Sum	GAPT	Diff
H_h_	–0.084	–0.227	0.020	–0.292	–0.295	0.003
H_p_	0.586	–0.210	0.028	0.404	0.405	–0.001
C_1_	0.819	–0.304	0.158	0.673	0.668	0.005
O_1_	–1.156	0.592	–0.122	–0.686	–0.680	–0.006
C_2_	0.818	–0.308	0.168	0.679	0.668	0.011
O_2_	–1.156	0.595	–0.126	–0.688	–0.680	–0.008

a All values are in units of electrons (e).

First of all, the reader is welcomed to check that the column containing the charge terms (C) in Table 2 is exactly equal to the respective charges appearing in Table 1, and the same can be said about GAPT in both tables. Therefore, we offer three different partition schemes for GAPT that start from different charge models but end up reproducing the very same GAPT parameter. In the case of ChelpG, the absence of atomic dipoles makes GAPT equivalent to a C+CT sum, whereas in the case of Hirshfeld and QTAIM, GAPT is built up by a C+CT+DP sum. One can see that the sums here match perfectly GAPT for all atoms, the differences being strictly null for CHELPG and Hirshfeld (for which the input values came from the same output file) whereas QTAIM models required an additional integration from AIMAll,^38^ thus inserting an additional numerical error that is nonetheless very small, not exceeding ± 0.01*e* in any case. The most important concept taken from Table 2 is that *the exact same set of GAPT parameters can be obtained using atomic charges that might vary in magnitude and even in sign*, since Hirshfeld charges are positive, whereas CHELPG and QTAIM charges are negative for the atoms therein tested. In other words, GAPT (usually called “APT charge”) *is not a charge precisely because it can be constructed from various different atomic charges by computing the changes in these charges upon vibration.* Indeed, Cho et al. reported a surprisingly high correlation between QTAIM charges and GAPT,^2^ and we explained the origin of this correlation based on the expected behavior of CT and DP derivatives.^14^

We do not aim to raise any dispute with Karton et al. about the various interesting insights they offered about this reaction and the possibilities of using it to model other catalyst-free protocols for hydrogen transfer. For example, the reaction barriers reported are not affected at all by the arguments we present here. Nonetheless, we aim to clarify the situation regarding GAPT and its relation to other charge models to avoid problems in identifying atoms that are really positively/negatively charged in molecules.

## Supplementary Material



## Notes

Tables of coordinates, CHELPG/CCT partition, Hirshfeld/CCTDP partition, QTAIM/CCTDP partition (PDF)
