# Supplementary material for: Comment on “Intramolecular Proton-Coupled Hydride Transfers with Relatively Low Activation Barriers”
Source: J Phys Chem A. 2025 Dec 12;129(51):11888–91. doi: 10.1021/acs.jpca.5c05958 (PMC12746444; doi:10.1021/acs.jpca.5c05958)
Supplement: Supplementary file 1 [file jp5c05958_si_001.pdf]

Supporting Information for “Comment on ‘Intramolecular Proton-Coupled Hydride Transfers with Relatively Low Activation Barriers’ by Karton et al.”

Vinicius Martinelli and Wagner Eduardo Richter*

Department of Chemistry, State University of Maringá. Maringá, PR, Brazil;

E-mail: werichter@uem.br

Table S1: *xyz* coordinates for **n1_HH**.
$\nu^{\ddagger} = -1654.8\ cm^{-1}$

C	1.175511	-0.636591	-0.405932
H	-0.004067	-0.293256	-1.181470
C	0.157946	-1.040830	0.657506
O	1.803681	0.473477	-0.211329
H	0.864120	1.143885	0.186853
C	-0.696404	0.065848	0.045271
O	-0.342813	1.279028	0.305985
H	1.587075	-1.371771	-1.103876
H	0.471666	-0.866409	1.687575
H	-0.253170	-2.040072	0.507561
H	-1.712219	-0.133485	-0.308782

Table S2: *xyz* coordinates for **n2_HH**
$\nu^{\ddagger} = -1574.2\ cm^{-1}$

C	1.338705	-0.109976	0.734053
H	-0.016689	-0.158519	0.989847
C	1.223001	1.217798	-0.051947
C	-0.232189	1.230263	-0.605316
O	1.575310	-1.195747	0.070590
H	0.572748	-1.334415	-0.578067
C	-0.854727	-0.091055	-0.104562
O	-0.604776	-1.176932	-0.762915
H	1.739604	-0.077280	1.757009
H	-0.271930	1.250911	-1.697583
H	-0.789760	2.087679	-0.228588
H	1.987575	1.231533	-0.833608
H	1.405746	2.068725	0.610988
H	-1.835217	-0.046384	0.390298

Table S3: *xyz* coordinates for **n1_HMe**
$\nu^{\ddagger} = -1324.8\ cm^{-1}$

C	1.230838	-0.603972	-0.403045
H	0.143236	-0.296733	-1.193893
C	0.201678	-1.005316	0.674679
O	1.891370	0.473053	-0.187882
H	0.776278	1.219511	0.251532
C	-0.683320	0.066642	0.075486
O	-0.305466	1.295506	0.302677
H	1.641989	-1.393699	-1.047391
H	0.527666	-0.820375	1.700083
H	-0.199407	-2.011871	0.545168
C	-2.109077	-0.159394	-0.335238
H	-2.731212	-0.133352	0.569139
H	-2.443594	0.633960	-1.005790
H	-2.322099	-1.133677	-0.808502

Table S4: *xyz* coordinates for **n2_HMe**
$\nu^{\ddagger} = -1510.8\ cm^{-1}$

C	1.361427	-0.122255	0.706932
H	0.035498	-0.166879	0.998679
C	1.215561	1.235962	-0.016018
C	-0.203205	1.206600	-0.632860
O	1.580688	-1.179600	-0.004593
H	0.497337	-1.321197	-0.609601
C	-0.878390	-0.075705	-0.066092
O	-0.648353	-1.178702	-0.717876
H	1.804254	-0.116354	1.715281
H	-0.192195	1.122749	-1.722985
H	-0.776738	2.100614	-0.372579
H	2.011517	1.344982	-0.758086
H	1.314760	2.052341	0.706594
C	-2.231402	0.033738	0.603063
H	-2.981589	0.272585	-0.161326
H	-2.503945	-0.919758	1.059583
H	-2.251527	0.827290	1.354047

Table S5: CHELPG/CCT partition for GAPT for **n1_HH**, **n2_HH**, **n1_HMe**, and **n2_HMe**. All values in units of *electrons*, (e).

**n1_HH**	C	CT	DP	Sum	GAPT	Diff
H_h	-0.116	-0.180	—	-0.295	-0.295	0.000
H_p	0.403	0.002	—	0.405	0.405	0.000
C_1	0.430	0.239	—	0.669	0.668	0.000
O_1	-0.536	-0.145	—	-0.680	-0.680	0.000
C_2	0.433	0.235	—	0.668	0.668	0.000
O_2	-0.537	-0.143	—	-0.680	-0.680	0.000
**n2_HH**	C	CT	DP	Sum	GAPT	Diff
H_h	-0.092	-0.201	—	-0.293	-0.293	0.000
H_p	0.376	-0.011	—	0.366	0.366	0.000
C_1	0.351	0.361	—	0.712	0.715	-0.002
O_1	-0.506	-0.143	—	-0.649	-0.649	0.000
C_2	0.366	0.350	—	0.716	0.715	0.001
O_2	-0.509	-0.139	—	-0.649	-0.649	0.000
**n1_HMe**	C	CT	DP	Sum	GAPT	Diff
H_h	-0.142	-0.197	—	-0.339	-0.340	0.001
H_p	0.397	-0.015	—	0.383	0.381	0.001
C_1	0.460	0.268	—	0.728	0.728	0.000
O_1	-0.582	-0.161	—	-0.743	-0.742	-0.001
C_2	0.604	0.171	—	0.775	0.770	0.005
O_2	-0.536	-0.125	—	-0.661	-0.661	0.000
**n2_HMe**	C	CT	DP	Sum	GAPT	Diff
H_h	-0.107	-0.236	—	-0.342	-0.343	0.000
H_p	0.369	-0.003	—	0.366	0.366	0.000
C_1	0.345	0.440	—	0.784	0.784	0.000
O_1	-0.520	-0.173	—	-0.693	-0.693	0.000
C_2	0.564	0.244	—	0.808	0.808	0.000
O_2	-0.542	-0.131	—	-0.673	-0.674	0.000

Table S6: Hirshfeld/CCTDP partition for GAPT for **n1_HH**, **n2_HH**, **n1_HMe**, and **n2_HMe**. All values in units of *electrons*, (e).

**n1_HH**	C	CT	DP	Sum	GAPT	Diff
H_h	0.009	-0.268	-0.036	-0.295	-0.295	0.000
H_p	0.114	0.242	0.049	0.405	0.405	0.000
C_1	0.099	0.426	0.144	0.668	0.668	0.000
O_1	-0.232	-0.338	-0.110	-0.680	-0.680	0.000
C_2	0.099	0.426	0.143	0.668	0.668	0.000
O_2	-0.232	-0.338	-0.110	-0.680	-0.680	0.000
**n2_HH**	C	CT	DP	Sum	GAPT	Diff
H_h	0.018	-0.292	-0.019	-0.293	-0.293	0.000
H_p	0.108	0.207	0.051	0.366	0.366	0.000
C_1	0.092	0.490	0.132	0.714	0.715	-0.001
O_1	-0.223	-0.320	-0.106	-0.649	-0.649	0.001
C_2	0.092	0.491	0.132	0.716	0.715	0.001
O_2	-0.223	-0.320	-0.106	-0.649	-0.649	0.001
**n1_HMe**	C	CT	DP	Sum	GAPT	Diff
H_h	0.001	-0.311	-0.029	-0.339	-0.340	0.001
H_p	0.119	0.220	0.043	0.382	0.381	0.001
C_1	0.073	0.496	0.158	0.728	0.728	-0.001
O_1	-0.284	-0.363	-0.096	-0.743	-0.742	-0.001
C_2	0.148	0.568	0.059	0.775	0.770	0.005
O_2	-0.191	-0.378	-0.092	-0.661	-0.661	0.000
**n2_HMe**	C	CT	DP	Sum	GAPT	Diff
H_h	0.015	-0.345	-0.013	-0.343	-0.343	0.000
H_p	0.107	0.211	0.048	0.366	0.366	0.000
C_1	0.083	0.574	0.127	0.784	0.784	0.000
O_1	-0.241	-0.355	-0.097	-0.693	-0.693	0.000
C_2	0.128	0.628	0.052	0.808	0.808	0.000
O_2	-0.216	-0.375	-0.083	-0.674	-0.674	0.000

Table S7: QTAIM/CCTDP partition for GAPT for **n1_HH**, **n2_HH**, **n1_HMe**, and **n2_HMe**. All values in units of *electrons*, (e).

**n1_HH**	C	CT	DP	Sum	GAPT	Diff
H_h	-0.084	-0.227	0.020	-0.292	-0.295	0.003
H_p	0.586	-0.210	0.028	0.404	0.405	-0.001
C_1	0.819	-0.304	0.158	0.673	0.668	0.005
O_1	-1.156	0.592	-0.122	-0.686	-0.680	-0.006
C_2	0.818	-0.308	0.168	0.679	0.668	0.011
O_2	-1.156	0.595	-0.126	-0.688	-0.680	-0.008
**n2_HH**	C	CT	DP	Sum	GAPT	Diff
H_h	-0.040	-0.269	0.015	-0.294	-0.293	0.000
H_p	0.592	-0.256	0.030	0.366	0.366	0.000
C_1	0.799	-0.228	0.141	0.711	0.715	-0.004
O_1	-1.143	0.594	-0.096	-0.645	-0.649	0.004
C_2	0.800	-0.234	0.130	0.695	0.715	-0.020
O_2	-1.143	0.595	-0.089	-0.637	-0.649	0.012
**n1_HMe**	C	CT	DP	Sum	GAPT	Diff
H_h	-0.095	-0.265	0.009	-0.351	-0.340	-0.011
H_p	0.614	-0.208	-0.024	0.383	0.381	0.001
C_1	0.857	-0.252	0.126	0.731	0.728	0.002
O_1	-1.168	0.530	-0.106	-0.745	-0.742	-0.003
C_2	0.776	-0.119	0.125	0.782	0.770	0.012
O_2	-1.178	0.570	-0.056	-0.663	-0.661	-0.003
**n2_HMe**	C	CT	DP	Sum	GAPT	Diff
H_h	-0.044	-0.326	0.037	-0.333	-0.343	0.010
H_p	0.597	-0.258	0.030	0.368	0.366	0.002
C_1	0.807	-0.155	0.128	0.781	0.784	-0.003
O_1	-1.146	0.561	-0.109	-0.695	-0.693	-0.002
C_2	0.780	-0.072	0.100	0.808	0.808	0.000
O_2	-1.158	0.587	-0.112	-0.683	-0.674	-0.009
